# Alcohol consumption and its correlation with medical conditions: a UK Biobank study

**DOI:** 10.3389/fpubh.2024.1294492

**Published:** 2024-05-22

**Authors:** Craig S. Mayer, Paul Fontelo

**Affiliations:** Lister Hill National Center for Biomedical Communications, National Library of Medicine, National Institutes of Health, Bethesda, MD, United States

**Keywords:** alcohol, dose–response, alcohol type, UK Biobank, statistical modal analysis

## Abstract

**Background:**

Alcohol consumption has been associated with the occurrence of many health conditions. We analyzed UK Biobank data to explore associations of various conditions to type and amount of alcohol consumed. UK Biobank is a large biomedical database providing information from UK participants, including lifestyle questionnaires and diagnosis data.

**Methods:**

Using UK Biobank, we examined the relationship between weekly alcohol consumption, alcohol type and the incidence of eight select conditions. We calculated counts of individuals consuming each type diagnosed with these conditions. To assess the effect of alcohol consumption on each condition’s prevalence, we used log-logistic regression models to generate dose–response models for each alcohol type.

**Results:**

The alcohol consumed included: red wine (228,439 participants), white wine (188811), beer (182648), spirits (129418), and fortified wine (34598). We observed increased condition prevalence with increasing amounts of alcohol. This was especially seen for chronic obstructive lung disease, cirrhosis of liver, hypertension, gastritis, and type 2 diabetes. Beer consumers showed higher prevalence for most conditions while fortified wine had the largest increases in incidence rates. Only white wine showed decreased incidence for acute myocardial infarction. In general, the prevalence of many conditions was higher among alcohol consumers, particularly for hypertension, 33.8%, compared to 28.6% for non-drinkers.

**Conclusion:**

Although many conditions were already prevalent among non-drinkers, participants consuming increasing amounts of alcohol had increased incidence rates for many of the studied conditions. This was especially true for consumers of beer and fortified wine, but also true to a lesser extent for consumers of spirits, red and white wine.

## 1 Introduction

Previous studies have explored the intake of alcohol and its negative effects on different organ systems and conditions (1, 2). These effects varied based on the quantity and types of alcohol consumed (3–5). In contrast, some other reports have expressed the positive effect of different alcohol types on certain medical conditions (6–8). There is also variability in the effects of alcohol on the occurrence of different medical conditions, as well as the impact and severity of the condition.

The UK Biobank program (UK Biobank) is a large health and biomedical database that serves multiple retrospective, observational studies and includes over half a million participants (9). UK Biobank contains questionnaire, diagnosis, procedures, drug, and lab measurement data for some or all of the participants depending on the data type and field.

As part of the data collection process, UK Biobank asked participants about their drinking habits including the amount and type of alcohol they consumed on a weekly or monthly basis. This data spans from 2012 to the present time, however, there are only a limited number of data points for each individual and the majority of participants may have provided only a single data point regarding their drinking habits. Despite the data from each participant being limited, the large number of participants in the study provides an informative representation of the types and quantities of alcohol consumed by the participants.

The alcohol types included in the questionnaire were beer and ciders (referred to as beer), red wine, white wine and champagne (referred to as white wine), spirits and fortified wine. Each alcohol type was measured differently: red, white, and fortified wine in glasses, beer in pints, and spirits in measures (~25 mL) (10). Each alcohol type includes a question regarding both weekly and monthly alcohol consumption. In our analysis, we focused on weekly alcohol consumption by type. UK Biobank also provides imported EHR data from hospital inpatient encounters using ICD-9 and ICD-10 codes, which were used to determine condition occurrences for the population.

## 2 Materials and methods

### 2.1 Alcohol type relationship to condition incidence

Our analysis examined the relationship between weekly alcohol consumption, alcohol type and the proportion of UK Biobank participants with certain medical conditions. These conditions included: acute myocardial infarction, cerebral infarction, chronic obstructive lung disease, cirrhosis of liver, congestive heart failure, essential hypertension, gastritis, and type 2 diabetes. Our assessment did not differentiate when these conditions occurred for an individual relative to their responses to alcohol consumption questions, only whether they were ever diagnosed with the studied condition in the provided data.

We calculated the number of individuals who reported consuming each alcohol type, including those who consumed only one or multiple alcohol types. We then calculated the percentage of individuals in UK Biobank who were diagnosed with these conditions in relation to the type of alcohol consumed.

### 2.2 Model fitting

To assess the effect of different quantities of alcohol consumed by type, we calculated the proportion of participants at that quantity and alcohol type with a condition compared to the overall number of participants who consumed that quantity of that type of alcohol. We analyzed changes in these proportions by changes in amount of alcohol consumed including changes in condition incidence rates between those who do not consume that alcohol type (zero-point) and those who consume each type of alcohol. In “zero-point” we refer to individuals who have indicated not consuming a particular type of alcohol. This is distinct from those who completely abstain from alcohol (non-drinkers).

We analyzed the dose–response effect of alcohol consumption on the proportion of individuals with each condition by using the *drm* R package and a log-logistic regression model (11). This approach provided dose–response (regression) curves for each alcohol type, showing the association between amount consumed and proportion of individuals with a specific condition at different quantities of alcohol consumed. Using the same *drm* R package, we calculated changes in condition incidence based on quantity consumed by alcohol type. This included the calculation of the statistical model coefficients relating to the regression analysis. The calculated coefficients indicate the direction of the model. A negative coefficient indicates an increase in condition incidence as the amount of alcohol consumed increases. A positive coefficient indicates the inverse. A *p*-value of less than 0.05 was used to determine whether the value indicated was statistically significant. We also determined the quantity of alcohol where a statically significant change (*p*-value <0.05) in response was found between alcohol quantities.

For individuals who consumed multiple types of alcohol, we calculated the correlation values (coefficient) between change in condition incidence rates while keeping the primary alcohol amount constant and increasing the amount of secondary alcohol consumed. We considered any value above 0.4 (*p*-value <0.05) as significant, indicating an increase in secondary alcohol amount shows an increase in condition incidence rates (12). We also performed multivariate analysis of all alcohol types simultaneously to understand their contribution to condition incidence rates in a single regression model.

## 3 Results

### 3.1 Population characteristics

Of the 502,390 UK Biobank participants, 344,410 (68.55%) reported consuming alcohol. Among the respondents, the consumption of alcohol by type included the following by participant: red wine (228439), white wine (188811), beer (182648), spirits (129418), and fortified wine (34598). Table 1 shows the demographics of each cohort of participants by alcohol type consumed.

**Table 1:** Demographics by alcohol type.

	Beer	White wine	Red wine	Spirits	Fortified wine
Participant count	182,648	188,811	228,439	129,418	34,598
Sex: male	142,021 (78%)	79,020 (42%)	117,754 (52%)	70,462 (54%)	14,881 (43%)
Sex: female	40,627 (22%)	109,791 (58%)	110,685 (48%)	58,956 (46%)	19,717 (57%)
Median age	57	58	59	60	61

*Numbers in parenthesis are percentages in each category.

There were distinctions in the gender distribution across various types of alcoholic beverages. Specifically, the proportion of male consumers varied significantly, ranging from 78% for beer to 42% for white wine. Red wine consumption rate among males was 52%, while spirits and fortified wine both were around 43%, showing a relatively balanced gender difference.

### 3.2 Multiple type consumers

Most individuals regularly consumed more than one type of alcohol on a weekly basis shown by the combined participant counts of each type being greater than the total participant count. In total, 247,649 (71.91%) of the 344,410 participants who stated they drink alcohol on a weekly basis, consumed more than one type. The most common combination of alcohol types consumed was red and white wine, reported by 136,287 participants.

### 3.3 Overall condition incidence

The incidence of different conditions varied between those who consumed different types of alcohol. Table 2 shows the percentage of individuals with a condition based on the type of alcohol. The table shows the population percentage with a condition for those who did not consume that type of alcohol (Zero) compared to the percentage of the population who consumed the largest quantity of that type of alcohol on a weekly basis (Max).

**Table 2:** Population percentage of participants at the zero-point (does not consume that alcohol type) and at the highest amount of alcohol consumed by type for each condition.

Condition	Beer		Red wine		White wine		Fortified wine		Spirits	
	Zero	Max	Zero	Max	Zero	Max	Zero	Max	Zero	Max
Acute myocardial infarction	0.8%	1.5%	1.3%	1.4%	1.5%	1.3%	1.2%	2.0%	1.1%	2.0%
Cerebral infarction	1.4%	1.8%	1.9%	2.5%	2.0%	2.1%	1.7%	2.6%	1.5%	3.7%
Chronic obstructive lung disease	2.9%	22.8%	5.8%	10.3%	5.1%	8.6%	3.8%	55.6%	3.3%	20.3%
Cirrhosis of liver	0.2%	4.1%	0.4	1.0%	0.4%	1.0%	0.3%	0.4%	0.3%	10.0%
Congestive heart failure	1.1%	4.1%	1.9%	1.9%	1.9%	2.3%	1.5%	2.0%	1.3%	4.3%
Essential hypertension	27.5%	49.1%	32.9%	64.3%	33.6%	40.7	30.1%	62.5%	28.2%	46.2%
Gastritis	7.4%	22.8%	8.9%	17.1%	8.2%	10.7%	7.4%	12.2%	7.0%	17.2%
Type 2 diabetes mellitus	5.0%	17.5%	8.3%	10.3%	8.3%	8.6%	6.7%	26.3%	5.8%	18.8%

As seen in Table 2, those who consumed red or white wine had a lower occurrence of each studied condition than consumers of any other type of alcohol as the increase in condition occurrence rate for red and white wine drinkers was the smallest for nearly every condition. Among the other alcohol types, beer, fortified wine, and spirits had the highest rate of incidence increases for those who consumed each, but differed based on the condition. Table 2 shows only the difference between non-drinkers of a particular type of alcohol and those who consume the maximum seen amount of alcohol. Below we discuss in depth the distribution based on varying amounts of alcohol consumed to show a more comprehensive analysis of the effects of different alcohol types and amounts on condition incidence rates.

### 3.4 Condition incidence by type and amount

**Figure 1:**
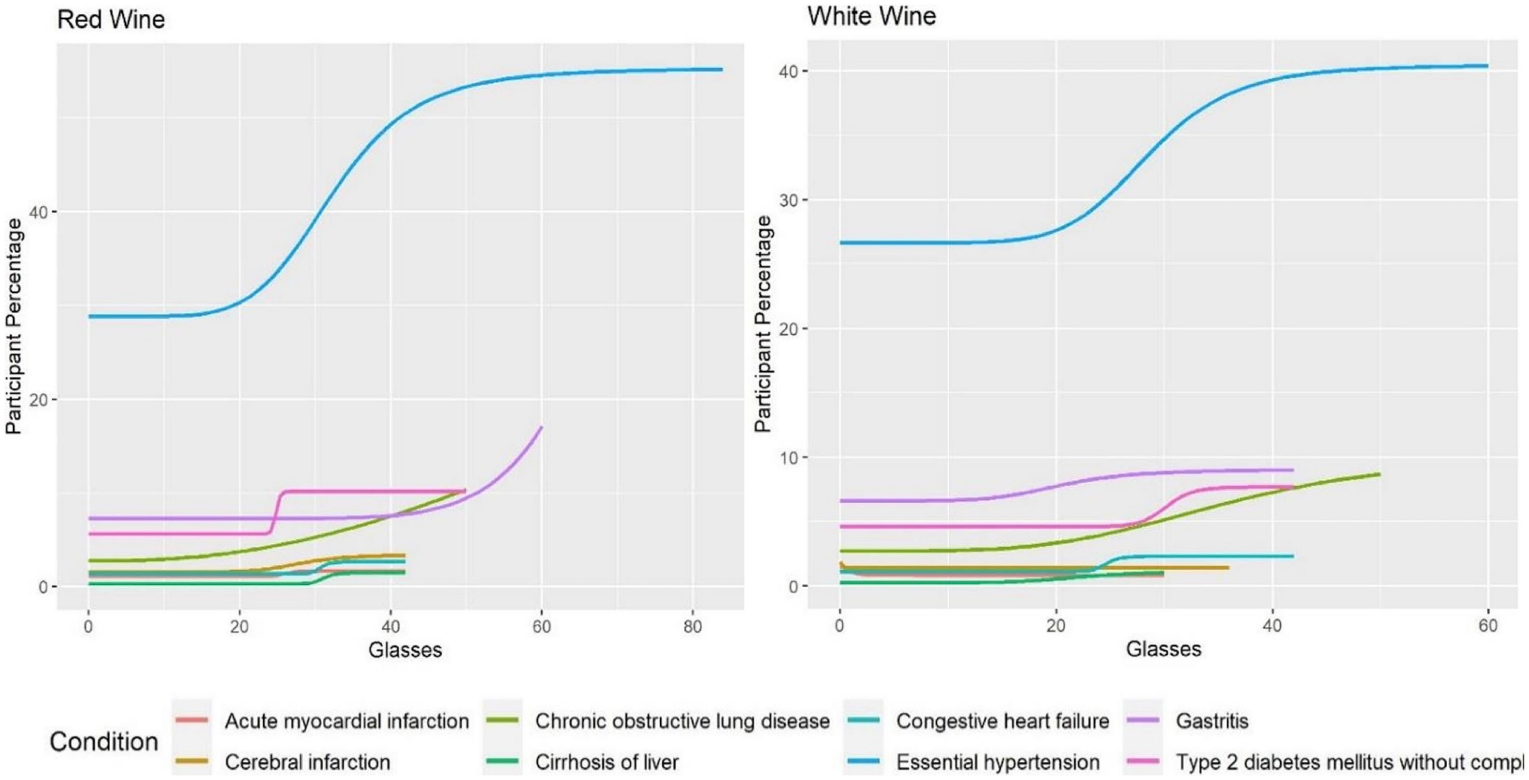
Amount of alcohol and participant percentage by condition for red and white wine.

While consuming one type of alcohol differed when compared to other types affected the incidence of different conditions, the rate of incidence for a given condition also varied based on the amount of each alcohol type consumed. Figure 1 shows the regression models for rate of condition occurrence by amount consumed for red and white wine.

Figure 1 shows similar trends between red and white wine for many of the conditions. The figure also shows the only decrease in condition occurrence found with increasing amounts consumed. This was seen in acute myocardial infarction where there were fewer participants with increasing consumption of white wine (coefficient: 2.77, *p*-value: 0.033). In comparison, red wine did not show a statistically significant change in occurrence of acute myocardial infarction with changes in amount consumed (coefficient: −29.54, *p*-value: 0.184). Red wine, except for an early rise, seemed to plateau at higher weekly quantities. The exceptions to this general trend for red wine were gastritis (coefficient: −8.38, p-value: 0.017) and chronic obstructive lung diseases (coefficient: −2.36, *p*-value: 0.029) which showed exponential increases.

**Figure 2:**
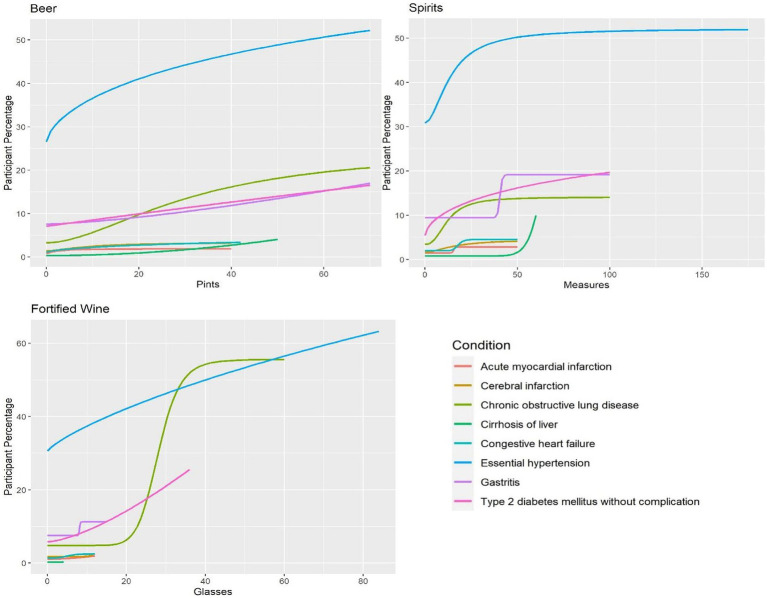
Amount of alcohol and participant percentage by condition for beer, spirits and fortified wine.

In Figure 2, the regression models show larger effects on condition occurrence rates for beer, fortified wine and spirits. In general, Figure 2 shows participants who consumed increasing quantities of beer on a weekly basis had an increased condition occurrence rate for nearly every condition. Fortified wine showed sharp increases in incidence rates for chronic obstructive lung disease (coefficient: −10.24, *p*-value: 0.0039), gastritis (coefficient: −75.68, *p*-value: 0.049) and essential hypertension (coefficient: −0.80, *p*-value: 0.026). Otherwise, the data is not sufficient to derive inference with regards to other conditions since no statistically significant trend was detected. Spirits showed the same increasing trend as beer and fortified wine, especially for essential hypertension (coefficient: −1.81, *p*-value: 0.044).

Overall, Figures 1, 2 show that consumption of higher quantities of alcohol was correlated with increased condition incidence rates for many of the studied conditions. Essential hypertension and chronic obstructive lung disease had the highest change observed among the alcohol types even with lower quantities, as shown by steep increases in incidence rates as higher amounts were consumed. In contrast, acute myocardial infarction, cerebral infarction, and congestive heart failure showed no steep increases with increasing alcohol consumption. A slight decrease in the incidence of acute myocardial infarction was seen only with white wine compared to other alcohol types and conditions.

In addition, we calculated the amount of alcohol consumed weekly, where we observed a steep and substantial change in incidence rate. Table 3 shows the quantity of alcohol where this occurred for each alcohol type and condition. In some cases, such as acute myocardial infarction for beer, fortified wine and white wine, and other conditions reviewed, this type of change was not seen.

**Table 3:** Trend change point by alcohol type and condition (value indicates amount of alcohol consumed weekly).

Condition	Beer (pints)	Fortified Wine (glasses)	Red Wine (glasses)	Spirits (measures)	White Wine (glasses)
Acute myocardial infarction			26.0 (<0.0001)	15.4 (<0.0001)	
Cerebral infarction			27.7 (<0.0001)		
Chronic obstructive lung disease	32.3 (0.047)	28.1 (<0.0001)		12.2 (<0.0001)	36.1 (<0.0001)
Cirrhosis of liver			31 (<0.0001)		22.0 (<0.0001)
Congestive heart failure		5.1 (0.0237)	30.7 (<0.0001)	17.8 (<0.0001)	24.6 (<0.0001)
Essential hypertension			32.4 (<0.0001)	13.9 (0.006)	28.6 (<0.0001)
Gastritis		8.1 (<0.0001)		40.5 (0.0001)	20.3 (0.008)
Type 2 diabetes mellitus			24.8 (<0.0001)		30.1 (<0.0001)

*Blank cells indicate no point could be derived for the condition and alcohol combination.

() Represents the *p*-value for the effective dose.

The point where the change in model trend occurred varied by alcohol type and condition. In many cases it occurred as higher amounts of alcohol are consumed. This is exemplified with red wine and type 2 diabetes where 24.8 glasses per week is shown as the change point. Only 1.5% of red wine consumers have more than 24 glasses on a weekly basis. For fortified wine and congestive heart failure this quantity was 5.1 glasses, which can be related to the high percentage of fortified wine consumers (10.7%) who drink at least five glasses per week and its higher alcohol content.

For individuals that drink multiple types of alcohol (247,649 individuals). Table 4 shows the effect that the addition of a second type of alcohol may have on condition incidence. The table includes the correlation values between increasing one type of alcohol (listed as the column headings) to those who already consume another type of alcohol (the row headings).

**Table 4:** Correlation coefficients between secondary alcohol consumption and change in condition incidence.

	Condition	Beer	Fortified wine	Red wine	Spirits	White wine
Beer	Acute myocardial infarction	x	0.43	**0.86**	**0.51**	**0.80**
	Cirrhosis of liver	x	**0.91**	**0.75**	**0.73**	**0.87**
	Essential hypertension	x	**0.69**	**0.77**	**0.56**	**0.72**
	Type 2 diabetes mellitus without complication	x	**0.75**	**0.71**	**0.46**	**0.45**
	Gastritis	x	**0.77**	**0.76**	**0.72**	**0.56**
	Cerebral infarction	x	**0.77**	**0.73**	**0.53**	**0.67**
	Congestive heart failure	x	**0.74**	**0.68**	**0.62**	**0.56**
	Chronic obstructive lung disease	x	**0.76**	**0.68**	**0.55**	**0.74**
Fortified wine	Acute myocardial infarction	0.32	x	**0.84**	**0.61**	0.47
	Cirrhosis of liver	**0.79**	x	**0.55**	**0.68**	0.21
	Essential hypertension	**0.65**	x	**0.51**	0.33	**0.45**
	Type 2 diabetes mellitus without complication	**0.77**	x	**0.77**	**0.55**	**0.81**
	Gastritis	**0.60**	x	**0.79**	**0.66**	**0.87**
	Cerebral infarction	**0.79**	x	**0.88**	**0.71**	**0.72**
	Congestive heart failure	**0.61**	x	**0.81**	**0.77**	**0.76**
	Chronic obstructive lung disease	**0.75**	x	**0.56**	**0.77**	**0.59**
Red wine	Acute myocardial infarction	**0.53**	**0.83**	x	**0.74**	**0.74**
	Cirrhosis of liver	**0.62**	**0.77**	x	**0.77**	**0.65**
	Essential hypertension	**0.84**	**0.48**	x	**0.48**	**0.67**
	Type 2 diabetes mellitus without complication	**0.90**	**0.80**	x	**0.53**	**0.73**
	Gastritis	**0.74**	**0.53**	x	**0.73**	**0.55**
	Cerebral infarction	**0.80**	**0.78**	x	**0.82**	**0.84**
	Congestive heart failure	**0.49**	**0.95**	x	**0.51**	**0.76**
	Chronic obstructive lung disease	**0.84**	**0.82**	x	**0.56**	**0.75**
Spirits	Acute myocardial infarction	**0.49**	**0.91**	**0.59**	x	**0.80**
	Cirrhosis of liver	**0.73**	**0.69**	**0.69**	x	**0.66**
	Essential hypertension	**0.62**	**0.51**	**0.61**	x	**0.65**
	Type 2 diabetes mellitus without complication	**0.64**	**0.95**	**0.57**	x	**0.55**
	Gastritis	**0.64**	**0.81**	**0.73**	x	**0.83**
	Cerebral infarction	**0.74**	**0.91**	**0.59**	x	**0.55**
	Congestive heart failure	**0.69**	**0.73**	**0.69**	x	**0.81**
	Chronic obstructive lung disease	**0.76**	**0.81**	**0.86**	x	**0.77**
White wine	Acute myocardial infarction	**0.60**	**0.63**	**0.70**	**0.81**	x
	Cirrhosis of liver	**0.49**	**0.84**	**0.76**	**0.83**	x
	Essential hypertension	**0.52**	**0.70**	**0.58**	**0.55**	x
	Type 2 diabetes mellitus without complication	**0.76**	**0.94**	**0.78**	**0.78**	x
	Gastritis	**0.69**	**0.82**	**0.61**	**0.55**	x
	Cerebral infarction	0.36	**0.91**	**0.70**	**0.77**	x
	Congestive heart failure	**0.69**	**0.69**	**0.79**	0.38	x
	Chronic obstructive lung disease	**0.77**	**0.80**	**0.62**	**0.72**	x

*Bolded values indicate a significant correlation (*p*-value < 0.05).

Table 4 demonstrates that for nearly every combination of alcohol types, there is an increase in the incidence rates (signified by a value greater than 0.4) of the studied condition. These findings corroborate the previously stated results, which showed that increases in the consumption of most alcohol types lead to higher overall incidence rates of the condition. Consuming different types of alcoholic drinks may have a compounded adverse effect on the body, potentially causing more harm than consuming only one type of alcohol.

We used the multivariate analysis for all five alcohol types to generate model coefficients for regression on all alcohol types simultaneously shown in Table 5 (bold cells are statistically significant, *p*-value<0.05).

**Table 5:** Multiple variable regression analysis model coefficients for all alcohol types.

	Red wine	White wine	Beer	Spirits	Fortified wine
Acute myocardial infarction	2.05E-05	**−0.00042**	**0.000594**	**0.000743**	−0.00106
Cirrhosis of liver	**7.93E-05**	**0.000187**	**0.000508**	**0.00045**	0.000342
Essential hypertension	**0.001024**	**−0.00293**	**0.00733**	**0.009288**	**0.016476**
Type 2 diabetes mellitus without complication	**−0.0009**	**−0.00261**	**0.002429**	**0.002978**	−0.00278
Gastritis	**−0.00054**	−0.0002	**0.001034**	**0.002503**	**0.004953**
Cerebral infarction	−0.00015	**−0.00043**	**0.000557**	**0.000526**	0.000454
Congestive heart failure	**−0.00017**	**−0.00033**	**0.000826**	**0.000759**	0.000599
Chronic obstructive lung disease	2.15E-05	0.000261	**0.003495**	**0.005216**	0.001773

*Bolded values indicate statistically significant value (*p*<0.05).

As shown in Table 5, using multiple variable regression analysis to examine the impact of five alcohol types simultaneously, we found that beer and spirits had the greatest effect on increasing the incidence rates for the studied conditions. The model coefficients for beer and spirits were statistically significant and positive, indicating their consumption contributes to higher incidence rates across all the examined conditions. In contrast, red wine had a statistically significant impact on the incidence rates for only five conditions, white wine for six conditions, and fortified wine for two conditions when all alcohol types were analyzed together. These findings suggest that while beer and spirits are associated with increased incidence rates for all the studied conditions, the effects of different wine varieties are more condition-specific when accounting for all alcohol types consumed by an individual.

## 4 Discussion

### 4.1 Overall trends

We undertook this study to determine the correlation of alcohol consumption on selected health conditions. Overall, for most conditions and alcohol types there was an adverse effect associated with increasing amounts of alcohol consumed shown by increases in condition incidence rates. These rising incidence rates were either seen as gradual at any increasing quantity consumed or as significant spikes following the consumption of substantial amounts of alcohol. One possible exception to this trend was observed with white wine and the occurrence of acute myocardial infarction where increasing amounts of alcohol may have led to a slight decrease or a steady rate for condition incidence.

While previous reports have expressed potential health benefits of red wine (6), our review indicates that higher consumption of red wine does not show any decreases in incidence rates for any of the conditions studied. Moreover, our results showed that increasing quantities of red wine may increase the occurrence of some of these conditions, although to a lesser extent than beer, spirits or fortified wine and commonly at higher amounts consumed on a weekly basis.

### 4.2 Non-drinkers

Of significance is the observation that even among individuals who completely abstain from alcohol consumption, zero-dose participants, the conditions under investigation are also frequently encountered. This outcome is not surprising, given the widespread nature of these conditions within the general population.

However, the study revealed the consistent correlation between the consumption of alcohol and the occurrence rate of these conditions. For example, this trend is particularly evident in the case of essential hypertension. Even among non-drinkers, the prevalence of essential hypertension is high, 28.6% however, among those who consumed any form of alcohol, this percentage increases to 33.8%. This may reflect on the common occurrence of essential hypertension in the participant community even with non-drinkers (also below).

### 4.3 Demographic affect

A recently released national health survey conducted by the NHS in 2021 showed a prevalence for hypertension of 30% among adults,15% of which are untreated (13). The study also showed hypertension increased with age from 9% among adults in the 16 to 44 age group to 60% in adults 65 years and older. Our UK Biobank data showing an average of 28.6% without the introduction of alcohol seems to agree with national statistics. The number of untreated hypertensives patients in our population is unknown.

The population enrolled in UK Biobank (average age of 57 years old) may have contributed to an increased incidence rate of certain conditions over what would be seen in the general public with a more even age distribution. This effect may also influence condition incidence for each alcohol type as the median age varied by alcohol type from 57 years old for beer to 61 years old for fortified wine.

Similar differences can be true depending on sex, as certain conditions are more common in one sex compared to the other. Previous research has shown conditions such as hypertension and cirrhosis of the liver are more prevalent in men than in woman (14, 15).The sex distribution varied greatly between alcohol types as exemplified by the difference in the populations of beer (77.76% male) and white wine (41.85% male) consumers. Previous studies have also shown that similar amounts of alcohol may have a different effect depending on the sex of the individual as large quantities may affect woman differently than men when it comes to condition occurrences and outcomes (16).

The occurrence of these conditions when no alcohol is consumed, especially hypertension, suggests public health measures of diagnosis and intervention for these conditions may be warranted.

### 4.4 Limitations

This assessment depicts a fragment of the relationship between alcohol and condition incidence and long-term studies are necessary to depict the complete impact of alcohol on condition occurrence. The data relied on participant responses to questionnaires – recall of events might be a limitation. This study is limited by variation in measurement units, alcohol content and the varying participant counts by type and amount consumed. The older population, consumption of multiple types and the UK based population limits the scope of the assessment, but it may still be generalizable to the global population. We did not disaggregate by gender within each alcohol type which may have affected the condition incidence rates although NHS data showed similar levels of overall prevalence of hypertension for both men and women (12).

For our assessment by alcohol type, we did not normalize by alcohol content. There is significant variability in alcohol content within and between each type of alcohol. In addition, while there are typical amounts of alcohol by glasses and measures (for wine and spirits), there may also be variability based on the measuring done, whether by what a consumer reports or by a beverage provider. Due to this variability, our analysis was done on provided measures by UK Biobank instead of by normalized amount. However, this does not change the effects shown; consuming increasing amounts of alcohol regardless of alcohol type resulted in an increased incidence rate of many of the studied conditions.

Due to the limited number of data points available for each individual, we were unable track a single individual or group over an extended period of time. The regression curves, especially the first and last points, although giving the impression of time progression, do not represent a sequence of time. Instead, the first and last points represent two distinct groups of people. This approach was adopted because our knowledge about participants’ alcohol consumption before entering the study is limited. Our focus is primarily on their current consumption patterns.

Furthermore, since our study did not assess additional risk factors associated with the studied conditions including other lifestyle factors, personal and family medical history, this could be a limitation.

## 5 Conclusion

We analyzed data gathered from the UK Biobank (UK Biobank) to examine the relationship between weekly alcohol consumption and the incidence of specific medical conditions. Across these conditions and types and quantities of alcohol a consistent pattern seems to emerge showing a higher correlation of incidence rates as alcohol intake increased. An exception was the slight decrease to stable incidence that was observed with white wine and acute myocardial infarction. Those who consumed fortified wine and beer showed the highest increases in incidence of the studied conditions with increasing amounts of alcohol. Further study is needed to characterize the effects of alcohol on different organ systems. The occurrence of many of these conditions, especially essential hypertension, in those who abstained from alcohol suggests that public health measures may be indicated.

## Data availability statement

The original contributions presented in the study are included in the article, further inquiries can be directed to the corresponding author.

## Ethics statement

Ethical approval was not required for the studies involving humans because this work was done as secondary research on de-identified data and is considered not-human subjects research according to the US National Institutes of Health IRB. The studies were conducted in accordance with the local legislation and institutional requirements. Written informed consent for participation was not required from the participants or the participants’ legal guardians/next of kin in accordance with the national legislation and institutional requirements because this work was secondary research.

## Author contributions

CM: Writing – review & editing, Writing – original draft, Visualization, Software, Resources, Project administration, Methodology, Investigation, Formal analysis, Data curation, Conceptualization. PF: Writing – review & editing, Writing – original draft, Visualization, Validation, Methodology, Investigation, Conceptualization.
